# A dataset of void characteristics in multidirectional carbon fiber/epoxy composite laminates, obtained using X-ray micro-computed tomography

**DOI:** 10.1016/j.dib.2019.104686

**Published:** 2019-10-18

**Authors:** Mahoor Mehdikhani, Ilya Straumit, Larissa Gorbatikh, Stepan V. Lomov

**Affiliations:** a KU Leuven, Department of Materials Engineering, Kasteelpark Arenberg 44, 3001, Leuven, Belgium; b SIM M3 program, Technologiepark 935, B-9052, Zwijnaarde, Belgium

**Keywords:** Voids, Porosity, X-ray computed tomography, Polymer-matrix composites

## Abstract

In the current data article, we present detailed characteristics of voids in carbon/epoxy composite laminates, along with the original image stacks obtained via X-ray micro-Computed Tomography (micro-CT)^1^. Five different lay-ups are produced with altering the recommended cure cycle in order to intentionally induce voids in the material. For each lay-up, an image stack (consisting of tomographic slices) and a dataset are provided. The image slices are in 8-bit TIF format. The datasets (spreadsheets) include the volume, size parameters, shape parameters, orientation, and location of the ellipsoids that are fitted to the detected voids in the specimen. The segmentation of the images and quantification of voids are performed in *VoxTex*, an in-house software for processing of micro-CT results. The processing and interpretation of the data is reported in [1]. The data is hosted in the *Mendeley Data* repository at [2].

**Table:** Specifications Table

Subject area	Materials science
More specific subject area	Polymer composites
Type of data	Table (Spreadsheet), 8-bit TIF image
How data was acquired	Micro-CT - *HECTOR* system [2] (*XWT 240-SE* micro-focus source from *X-RAY WorX* and 40 × 40 cm^2^*PerkinElmer 1620 CN3 CS* flat panel detector)
Data format	Filtered (to exclude artefacts)
Experimental factors	Voids are deliberately induced in laminates with different lay-ups via altering the recommended cure cycle.
Experimental features	Specimens are cut from the laminates and scanned using micro-CT with a resolution of 6.56 μm/pixel to characterize the voids.
Data source location	Leuven, Belgium
Data accessibility	Accessible on the Mendeley Data repository at [2]
Related research article	Mehdikhani et al. Detailed characterization of voids in multidirectional carbon fiber/epoxy composite laminates using X-ray micro-computed tomography. Comp Part A. **125**, 2019,105532.

**Table:** 

**Value of the Data** • The data corresponding to characteristics of voids in carbon/epoxy laminates is reported in detail, which can be used, for example, as input for studies modeling similar composites with voids. To the authors' best knowledge, such detailed data does not exist in the literature despite a significant need for it. • The data allows statistical analysis of void characteristics, i.e. size, shape, orientation, and spatial distribution. Although the voids in this study are deliberately induced and, thus, excessive, natural voids in the same types of materials should have analogous shape and orientation distributions since these characteristics are governed by the microstructure. • Formation of voids in this type of material and the effect of stacking sequence on void characteristics can be realized by investigating the data.

## 1 Data

The significance of voids and their characterization in fiber-reinforced composites are broadly reviewed in Ref. [3]. In this regard, voids in five carbon/epoxy laminates with different stacking sequences are investigated. The reconstructed micro-CT slices of representative specimens, cut from the laminates, are segmented to identify voids. The images in 8-bit TIF format are stored in Mendeley Data at [2]. The micro-CT resulting void content in the [±45]_2s_, [±67.5]_2s_, [67.5/22.5]_2s_, [0/90]_2s_, and [0/90]_4s_ specimens is 0.22%, 0.40%, 0.78%, 0.26%, and 1.24%, respectively. Although the void content values are quite small, the number of voids in each specimen is large enough to create reliable statistics.

**Fig. 1:**
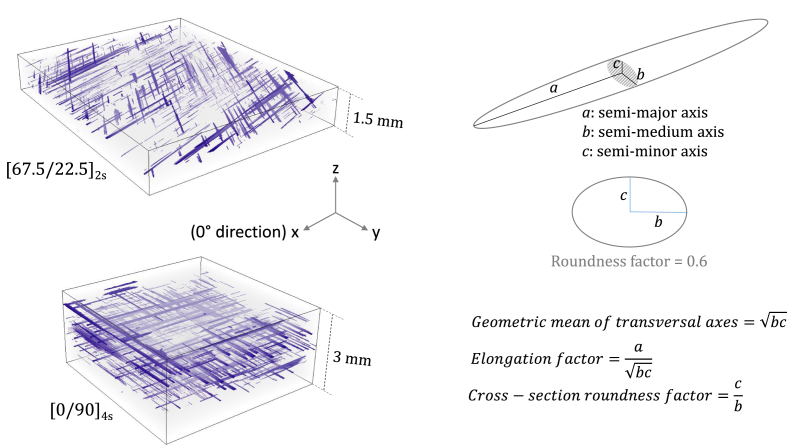
Left: 3D visualization of voids (in blue) detected via micro-CT in two specimens (as examples) – gray represents the volume of investigation; right: schematic of an equivalent fitting ellipsoid used for quantification of void characteristics and of an elliptic cross-section with a roundness factor of 0.6, along with the definition of the size and shape parameters.

For analysis of void characteristics, the voids are fitted to equivalent ellipsoids, as shown in Fig. 1 and explained in the following section. For each specimen, a dataset of characteristics of the ellipsoids fitted to the detected voids is reported in a separate data file, named with the stacking sequence of the corresponding laminate, e.g. “[+-45]2s.xlsx”. The data file of each specimen includes two spreadsheets, presented in two tabs. The first one, called “Void characteristics”, includes the volume, location, size parameters, in-plane orientation, and shape parameters of all detected voids in that specimen. Each row corresponds to one detected void, and the description of the columns is as follows.

• Column 1: measured volume of the actual void
• Column 2, 3, and 4: respectively x-, y-, and z-coordinates of the equivalent ellipsoid's centroid, where they correspond to the length (laminate's 0° direction), width (laminate's 90° direction), and thickness (laminate's out-of-plane direction) directions, respectively (see Fig. 1)
• Column 5, 6, 7: respectively semi-major, semi-medium, and semi-minor axes of the equivalent ellipsoid (see Fig. 1)
• Column 8: geometric mean of the transversal semi-axes, i.e. semi-medium and semi-minor axes (see Fig. 1)
• Column 9: in-plane orientation of the equivalent ellipsoid, defined as the angle between its major axis and the scan orientation, i.e. close (within few degrees) to the 0° direction of the laminate – after being calculated in *VoxTex*, the orientation is transformed to fall in [-90° 90°] for the [±45]_2s_ and [±67.5]_2s_ specimens and in [-45° 135°] for the [67.5/22.5]_2s_, [0/90]_2s_, and [0/90]_2s_ specimens.
• Column 10: elongation factor, which is the ratio of the major axis and the geometric mean of the transversal axes (see Fig. 1)
• Column 11: cross-section roundness factor, i.e. the ratio of the minor and medium axes (see Fig. 1)

The second spreadsheet, called “Statistics”, includes the results of a statistical analysis on columns 1, 5, 6, 7, 8, 10, and 11 of the “Void characteristics” spreadsheet. The analysis includes calculation of the mean, standard deviation, coefficient of variation, skewness, minimum, maximum, and 50th and 95th percentiles,^2^ of which the results are reported in different rows. The data can be accessed at the Mendeley Data repository at [2]. The processing and interpretation of the data is reported in Ref. [1].

## 2 Experimental design, materials, and methods

### 2.1 Composite laminates

Micro-CT characterization of voids is performed on a carbon/epoxy composite made from unidirectional prepreg tapes. The prepreg is made of high-strength standard-modulus carbon fibers, *Tenax® - E HTS40 F13 12K*, impregnated with a toughened epoxy resin, *CYCOM® 977-2*. Lay-ups are produced with automated tape laying and cured in an autoclave, both at *SABCA Limburg NV, Belgium*. In order to induce voids in the laminates (for void characterization), a low-pressure-temperature cure cycle, similar to Ref. [5], is followed instead of the manufacturer's cure cycle. The cycle includes curing at 150 °C and 0.5 bar followed by post-curing, while the manufacturer's cure cycle includes curing at 180 °C and 6 bar before post-curing. Debulking prior to cure and vacuum during cure are applied. The effect of defects on damage development in this material was studied in Refs. [6,7].

Five lay-ups are produced: [±45]_2s_, [±67.5]_2s_, [67.5/22.5]_2s_, [0/90]_2s_, and [0/90]_4s_, based on the ply degradation meso-model described in Ref. [8]. Specimens with the size of ∼15 mm × 10 mm × t are cut from the inner area of the produced plates, where t ≈ 1.4 mm for the 8-ply lay-ups and ≈ 2.8 mm for the 16-ply lay-up. Micro-CT analysis of a similar material, but produced with manufacturer's recommended cure cycle, does not reveal any voids, as investigated in Ref. [9].

### 2.2 Micro-CT image acquisition and processing

Micro-CT imaging was performed with the *HECTOR* system [4], which is from *Ghent University Centre for X-ray Tomography* (*UGCT*). The system has an *XWT 240-SE* micro-focus source from *X-RAY WorX* and a 40 × 40 cm^2^ *PerkinElmer 1620 CN3 CS* flat panel detector. Specimens were mounted and centered on the scanner rotation stage. The scanning volume was 12 × 12 × 12 mm^3^, and the resolution was 6.56 μm/pixel. The rotation increment was 0.15°, with 1 s exposure time for each projection, and the scan voltage and power were respectively 80 kV and 10 W. The reconstruction of the X-ray projections to tomographic slices was executed with the *Octopus* reconstruction software [10].

The segmentation and data processing are performed in the *VoxTex* software [11], which was developed for processing of micro-CT data in the Department of Materials Engineering (MTM), KU Leuven. The segmentation is based on the gray value of the images and is carried out using the Gaussian mixture model and the “expectation-maximization” algorithm. The latter needs definition of a “minimum void classification confidence”, which is set to 95% for the current analysis. For details of segmentation method, see Ref. [1].

In each image stack corresponding to each specimen, a region of interest covering the whole processable volume of the stack is selected. Therefore, small boundary regions are excluded from the analysis, resulting in slightly different sizes of region of interest for different specimens. A threshold of two voxels for the minimum void volume is defined, meaning that only voids equal to or larger than two voxels, i.e. 2 × 6.55^3^ = 562 μm^3^, are taken into account. The two-voxel threshold is the lowest possible value in the software. After segmentation, voids are fitted in *VoxTex* to equivalent ellipsoids with the same inertia tensor^3^ as that of the void. For each ellipsoid, the minor, medium, and major axes as well as the location of its centroid and the orientation are calculated (see Fig. 1 and the Data Section for the definitions).

**Fig. 2:**
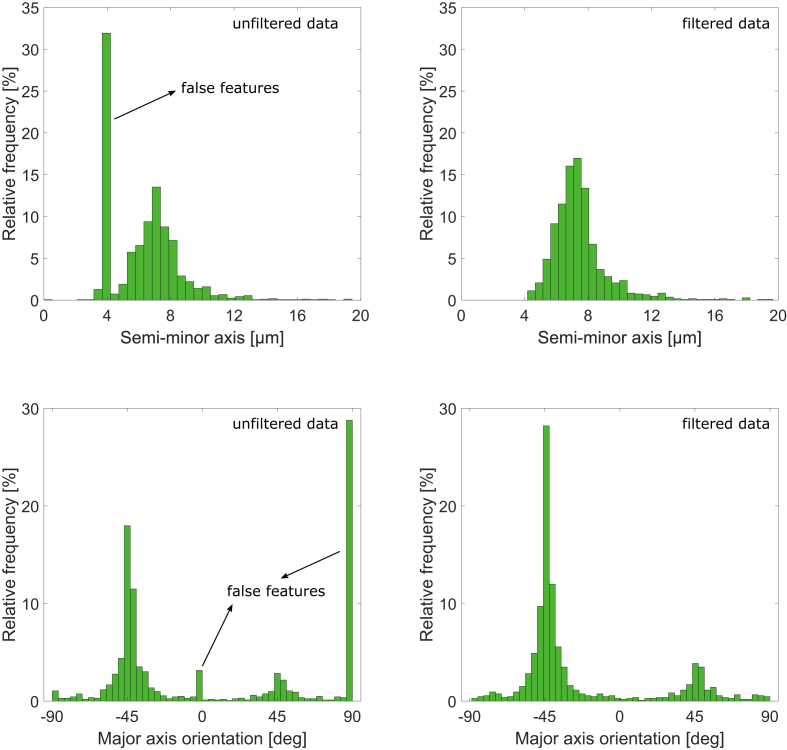
Frequency distributions of the semi-minor axis and the in-plane orientation of the ellipsoids fitted to the detected voids in the [±45]_2s_ specimen (left) before and (right) after filtering [1].

There are features detected with a semi-minor axis below 4.15 μm (minor axis of 8.3 μm), which are identified as “false voids” as they create non-physical clusters in the relative frequency distribution of the semi-minor axis and void orientation, as shown in Fig. 2 for the [±45]_2s_ specimen, as an example. Another reason to support that they are falsely-detected features is that their calculated orientation is an exact integer, like 0 or 90, which is not the case for the real voids. Therefore, these features are considered to be the noise created by micro-CT acquisition, reconstruction, or processing. They are filtered out and further analysis is performed without them. Moreover, detected features with semi-medium axis above 900 μm are filtered out because they are too large to be voids. They can be delaminations or flat cracks created during the preparation of small specimens for micro-CT. Note that in Fig. 2, the change that occurred to the rest of the histogram, after filtering the false features, is caused by recalculating the “relative” frequencies for the new data.

## Footnotes

2 The small difference in the percentile values between the spreadsheets and the reference article [1] is due to the difference in percentile calculation methods between *MATLAB* and *Microsoft Excel*.

3 A tensor consisting of nine components *I*_*ij*_, where on the main diagonal *i = j*, and elsewhere *i ≠ j* (*i,j: x,y,z*). *I*_*ii*_ is the moment of inertia about the *i*-axis, and *I*_*ij*_ is the *ij* product of inertia.
